# Comparison of Four Tests for the Diagnosis of *Helicobacter pylori* Infection

**DOI:** 10.3390/healthcare12151479

**Published:** 2024-07-25

**Authors:** Lior Charach, Tsachi Tsadok Perets, Rachel Gingold-Belfer, Yair Huta, Olga Ashorov, Zohar Levi, Ram Dickman, Doron Boltin

**Affiliations:** 1 Internal Medicine B, Rabin Medical Center, Petah Tikva 49100, Israel; liorch@clalit.org.il; 2 Gastroenterology Laboratory, Rabin Medical Center, Petah Tikva 49100, Israel; kaiser1974@gmail.com (T.T.P.); yairhu@clalit.org.il (Y.H.); olgaas@clalit.org.il (O.A.); 3 Department of Digital Medical Technologies, Holon Institute of Technology, Holon 58444, Israel; 4 Division of Gastroenterology, Rabin Medical Center, Petah Tikva 49100, Israel; rgb5895@gmail.com (R.G.-B.); zohar.levi.gastroenterology@gmail.com (Z.L.); dickmanr1@gmail.com (R.D.)

**Keywords:** *Helicobacter pylori*, 13C-urea breath test, stool test, rapid urease test

## Abstract

Background: Due to lower operational costs, health maintenance organizations (HMOs) may prioritize *Helicobacter pylori* stool antigen testing (HpStAg) for the non-invasive diagnosis of *H. pylori* infection over 13C-urea breath tests (13C-UBTs). The aim of our study was to compare the accuracy of the diagnostic tests for *H. pylori*. Methods: We performed histology, rapid urease test (RUT), 13C-UBT and HpStAg on consecutive patients referred for gastroscopy. Monoclonal stool antigen test was performed using the LIAISON Meridian chemiluminescent immunoassay. Histology was examined with hematoxylin and eosin, and additional stains were performed at the pathologist’s discretion. For the assessment of 13C-UBT, we compared concordant histology and RUT. HpStAg was compared to the concordant results of two of the three remaining tests. Results: 103 patients were included (36 males (35.0%), age 50.1 ± 18.4 years). The indication for gastroscopy was dyspepsia in 63 (61.2%). Agreement between RUT and histology was 95.9%. For 13C-UBT and HpStAg, respectively, *H. pylori* positivity was 30% (30/100) and 27.16% (22/81); sensitivity was 97% and 70%; specificity was 100% and 94.4%; accuracy was 98% and 86%; positive predictive value (PPV) was 100% and 86.4%; negative predictive value (NPV) was 93% and 86%. No demographic, clinical, or endoscopic predictors of HpStAg accuracy were identified using logistic regression. Conclusions: 13C-UBT performs better than HpStAg at our institution. When interpreting results, clinicians should consider test limitations.

## 1. Introduction

*Helicobacter pylori* (*H. pylori*) is a Gram-negative, spiral microaerophilic, flagellated bacteria infecting approximately half of the world’s population and is associated with the development of peptic ulcer disease (PUD), gastric adenocarcinoma and mucosa-associated lymphoid tissue (MALT) lymphoma [1,2,3,4,5].

Several tests are available for diagnosis of *H. pylori* infection, including endoscopy-based tests and tests which do not require endoscopy. Endoscopy-based tests include tissue histology (with or without specialized staining and immunohistochemistry), molecular tests, the rapid urease test (RUT) and tissue culture. Non-endoscopic tests include the 13C-urea breath test (UBT), *H. pylori* stool antigen test (HpStAg) and serological tests [6,7,8,9].

Histology may be considered the de facto gold standard for the diagnosis of *H. pylori*, with a sensitivity and specificity of 91–93% and 100%, respectively [10]. RUT is a rapid test in which a biopsy of gastric tissue is placed onto a medium containing urea and a phenol red pH indicator and utilizes the urease-secreting property of the organism. RUT has the added advantage of lower costs, since pathology services are not required [10]. However, for both histology and RUT, the need for endoscopy to obtain gastric tissue is a limiting factor, as endoscopy may not otherwise be indicated, particularly when testing to confirm eradication following treatment [10,11].

When choosing a test for the diagnosis of *H. pylori*, the clinician should take into account patient factors, administrative factors and epidemiological factors. Examples of patient factors include a test’s acceptability and personal preference (particularly pertaining to stool tests), whether symptoms are present which warrant esophagogastroduodenoscopy (EGD), whether the patient is pre- or post-treatment and whether the patient has experienced a previous treatment failure. Epidemiological factors include the pre-test probability of a positive result, the background prevalence of *H. pylori* and the accuracy of the chosen test in the particular population. Administrative factors include test availability and cost.

Over the past years, there has been a gradual worldwide shift from 13C-UBT to HpStAg as the non-invasive test of choice due to perceived administrative advantages, such as fast point-of-care collection and turnaround, as well as overall reduced costs [11,12]. However, there is concern that the accuracy of HpStAg, compared to 13C-UBT, is reduced [13,14].

The present study aims to evaluate the relative accuracy of the diagnostic tests for *H. pylori* infection at our institution.

## 2. Materials and Methods

### 2.1. Patients

We prospectively recruited subjects over the age of 18 years who presented to our institution between 1 January 2021 and 31 December 2022 for EGD, for any indication. Exclusion criteria included history of gastric and intestinal surgery, exposure to an antibiotic or bismuth in the past one month, exposure to a proton pump inhibitor (PPI) in the past 2 weeks, bleeding dyscrasias, active malignancy, severe systemic disease precluding treatment of *H. pylori* if detected and inability to provide informed consent.

### 2.2. Sample Acquisition and Analysis

#### 2.2.1. 13C-Urea Breath Test

Patients underwent UBT 1 h prior to EGD. The test meal consisted of 75 mg of 13C-urea and 4 g of citric acid dissolved in 100 mL of water [15]. The Gilson XL222 Automatic Breath Sampler (Gilson, Middleton, WI, USA) and AP2003 Isotope Ratio Mass Spectrometer (IRMS) (Analytical Precision, Phoenix, AZ, USA) were used to analyze breath samples. The ratio of expired 13C and 12C (parts per thousand) was recorded prior to and 30 min after ingestion of the test meal. The difference between these two scores, or the delta over baseline (DOB), constituted the final result. As per the manufacturer’s specifications, a result greater than 3.5 DOB was considered positive for the presence of *H. pylori*.

#### 2.2.2. Gastric Biopsy

Two biopsies from the gastric antrum, one from the incisura angularis and two from the gastric corpus were obtained and preserved in formalin. Paraffin blocks of biopsy materials were sectioned and stained with hematoxylin and eosin and histologically examined. Giemsa, Warthin–Starry and immunohistochemical stains were performed at the pathologist’s discretion. Gastritis was graded using the Updated Sydney System which assigns a score of 0, 1, 2 or 3 (corresponding to absent, mild, moderate and severe, respectively) to 5 parameters [16]. These parameters are chronic inflammation (lymphocyte and plasma cell infiltrate), acute inflammation (neutrophilic infiltrate), glandular atrophy, intestinal metaplasia and *H. pylori* colonization. The mean of the individual subscores for the corpus and antrum constituted the final score for each parameter in each subject.

In addition, one biopsy from the gastric antrum and one from the gastric corpus were obtained for RUT (Pronto dry, Gastrex, Brignais, France). The RUT result was recorded after 60 min according to the manufacturer’s specifications.

#### 2.2.3. *H. pylori* Stool Test

Following EGD, patients remained under observation in the recovery room for 1 h. During this time, patients provided a stool sample. Patients who were unable to provide a same-day sample were asked to return a sample within 3 days. A monoclonal stool antigen test was performed using the LIAISON Meridian chemiluminescent immunoassay (DiaSorin SpA, Saluggia, Italy). This is the test used by our institution and by the major HMOs in our country.

Subjects with *H. pylori* infection were prescribed treatment according to current guidelines [17].

The study was conducted in accordance with the principles of the Declaration of Helsinki and Good Clinical Practice (GCP) and was approved by the Human Subjects Protection Program of CHS (#RMC-20-0900).

#### 2.2.4. Statistical Analysis

SAS Software, version 9.4, was used for the statistical analysis. Categorical variables were expressed as N (%). Continuous variables were expressed as mean ± standard deviation. Categorical variables were compared using Fisher’s exact test (for two values) or the chi-square test (for more than two values). Continuous variables were compared using the *t*-test. Test performance was expressed as sensitivity (true positive (TP)/(TP + false negative (FN))), specificity (true negative (TN)/(TN + false positive (FP))), accuracy ((TP + TN)/(TP + TN + FP + FN)), positive predictive value (PPV) (TP/(TP + FP)), negative predictive value (NPV) (TN/(FN + TN)), positive likelihood ratio (sensitivity/(1 − specificity)) and negative likelihood ratio ((1 − sensitivity)/specificity).

For the assessment of histology, we compared concordant RUT and 13C-UBT (non-concordant data sets were excluded from the analysis). For RUT, the comparison was carried out with concordant histology and 13C-UBT. For the assessment of 13C-UBT, the RUT and histology were used for comparison. For the assessment of HpStAg, the comparison was carried out with at least two of the three remaining tests (RUT, histology and 13C-UBT). PPI exposure was measured in omeprazole equivalents [18].

Logistic regression was used to generate univariate and multivariate odds ratios (OR). Two-sided *p* values less than 0.05 were considered statistically significant.

## 3. Results

### 3.1. Demographic and Clinical Findings

A total of 103 patients were enrolled in this study, including 36 (34.95%) males, aged 50 *±* 18.3 years. Patient characteristics are summarized in Table 1. Overall, 59 patients (57.28%) were born in Israel, and 40 (38.83%) patients were current or past smokers. The most common indication for EGD was dyspepsia, followed by gastroesophageal reflux (63 (61.17%) and 19 (18.45%) subjects, respectively). Of the subjects, 64 (62.10%) had not previously received treatment for H. pylori infection (treatment-naïve).

### 3.2. Endoscopy Findings

Endoscopic findings are summarized in Table 2. Macroscopically, EGD was normal in 49 (47.50%) patients. Pangastritis was found in 17 (16.50%) subjects, isolated corporal gastritis in 6 (5.80%) subjects and isolated antral gastritis in 18 (17.50%) subjects. Peptic ulcer was found in 3 (2.87%) patients. Histological findings categorized according to Updated Sydney Classification are shown in Table 2.

### 3.3. H. pylori Positivity

*H. pylori* was positive by histology in 33/103 (32.04%), by rapid urease test in 35/98 (35.71%), by 13C-UBT in 30/100 (30.0%) and by HpStAg in 22/82 (26.82%).

### 3.4. Diagnostic Test Performance

The performance of each of the diagnostic tests for H. pylori is shown in Table 3. RUT and histology were discrepant in 4 cases. RUT and 13C-UBT were discrepant in 3 cases. 13C-UBT and histology were discrepant in 2 cases. For histology, RUT, 13C-UBT and HpStAg, respectively, test sensitivity was 100%, 100%, 97% and 70.4%; specificity was 98.4%, 96.8%, 100% and 94.4%; PPV was 96.8%, 93.8%, 100% and 86.4%; NPV was 100%, 100%, 98.4% and 86.4%; positive likelihood ratio was 62, 31.5, undefined and 18.65; negative likelihood ratio was 0, 0, 0.03 and 0.31. Logistic regression did not identify any demographic, clinical, or endoscopic predictors for HpStAg accuracy.

## 4. Discussion

Several tests are available for the diagnosis of *H. pylori*, with 13C-UBT and HpStAg being the preferred non-invasive tests, and histology and RUT the most common endoscopy-based tests [6,7,8,9]. In many HMOs worldwide, there has been a gradual shift from 13C-UBT to HpStAg due to lower operational costs. In the present study, we found that, at our institution, 13C-UBT has a superior sensitivity, specificity, PPV, NPV and accuracy, compared to HpStAg. This is the first study in our geographical region to assess the performance of HpStAg. Local validation of HpStAg is important since *H. pylori* prevalence varies greatly according to geographical region, and test characteristics such as PPV and NPV are dependent upon disease prevalence.

One of the main challenges in assessing the accuracy of a diagnostic test for *H. pylori* is the lack of a gold standard. Most previous studies compare HpStAg to a single test, for example, 13C-UBT or histology, both of which may be associated with false negative and false positive results [19,20,21,22,23,24]. Very few studies have a rigorous methodology such as we used, where subjects underwent four different tests on the same day, to create a de facto gold standard.

A notable exception is the landmark study performed by Monteiro et al., in which each subject underwent eight diagnostic tests (HpStAg, 13C-UBT, RUT, histology, serology, culture, immunoblot and PCR). The authors found that HpStAg had a sensitivity and specificity of 0.89 and 0.94, respectively, and PPV and NPV were both 0.91, lower than any of the other seven tests. In our study, the overall performance of HpStAg was inferior to the finding of Monteiro et al., who published their findings over two decades ago when HpStAg was mainly used in a research setting [25].

Our findings are consistent with previous studies. A Cochrane review by Best et al. found that assuming a fixed specificity of 0.90, 13C-UBT sensitivity was 0.94 with 30 missed *H. pylori* cases per 1000 tested [12]. In contrast, HpStAg sensitivity was 0.83 with 89 missed *H. pylori* cases per 1000 tested. However, these data are based on mainly indirect comparisons of highly heterogeneous studies. Altogether, only seven studies were identified which directly compared 13C-UBT and HpStAg, and these studies were considered generally of poor methodological quality.

A large meta-analysis by Zhou et al. found a better performance of HpStAg in children, with 0.96 sensitivity and 0.95 specificity for monoclonal HpStAg, and 0.88 sensitivity and 0.93 specificity for polyclonal HpStAg [26]. However, in the individual studies included, different reference tests were used, study heterogeneity was high, and publication bias was assessed to be high, which raises a suspicion of potentially inflated estimates due to poor study design. Our present study compares HpStAg to 13C-UBT, RUT and histology, which were all performed in every subject on the same day. Given the rigorous methodology used in our study, we believe that the lower estimation of HpStAg sensitivity and specificity which we found is more indicative of true test performance.

The Maastricht VI guideline recommends using either 13C-UBT or HpStAg for the primary diagnosis of *H. pylori* and for post-treatment confirmation of eradication [17]. Older, polyclonal stool antigen tests are considered less accurate than monoclonal tests and should be avoided. Similarly, the Maastricht VI guideline recommends using enzyme immunoassay (EIA) test kits rather than rapid immunochromatography tests, which should be abandoned [15,27,28,29,30]. Most importantly, the guideline recognizes the potential for low HpStAg test sensitivity and emphasizes that clinicians understand this limitation.

Although monoclonal stool antigen tests have been previously compared to polyclonal tests, and EIAs have been compared to rapid immunochromatography and lateral flow assays, there are no, large, head-to-head studies which compare the performance of the commercially available monoclonal immunoassays for *H. pylori* diagnosis.

Indeed, reliance on HpStAg may lead to missed infection on the one hand and unnecessary antibiotic exposure on the other. According to our data, for every 1000 HpStAg tests performed, 136 cases will be missed. These patients would be denied treatment and continue to harbor *H. pylori*. By comparison, when using 13C-UBT, only 16 cases per 1000 tests would be missed. On a population scale, a high false negative rate has the potential to adversely impact morbidity and gastric cancer incidence. On the flipside, for every 1000 HpStAg tests performed, 95 cases would be misclassified as positive, and unnecessarily be prescribed quadruple antibiotic therapy, as per current guidelines. For C13-UBT, however, no false positive tests were recorded. Our study shows that 13C-UBT has a comparable diagnostic performance to histology and a slightly higher accuracy than RUT.

The main strength of our study is its robust design. We prospectively enrolled patients to undergo at least four different diagnostic tests for *H. pylori*, thereby avoiding indirect comparisons and creating a de facto gold standard for *H. pylori* status, to which individual tests could be assessed. A potential limitation of our study is the lack of a post-treatment subgroup in order to assess non-invasive tests’ accuracy in eradication verification.

## 5. Conclusions

13C-UBT should be considered the non-invasive test of choice for *H. pylori* diagnosis at our institution. Despite the appeal of fast point-of-care collection, lower operational cost and rapid turnaround, the performance of HpStAg is inferior to that of 13C-UBT, histology and RUT. It is important for clinicians to be aware of these limitations when interpreting tests results.

## Figures and Tables

**Table 1:** Demographic and patient characteristics.

	N (%)
Total	103
Age, y, mean (SD)	50 ± 18.3
Sex, male	36 (34.95)
Ethnicity	
Ashkenazi	41 (39.81)
Sephardi	46 (44.66)
Arab	6 (5.83)
Other	10 (9.71)
Country of Birth	
Israel	59 (57.28)
Former Soviet Union (FSU)	17 (16.50)
Ethiopia	6 (5.83)
Middle East	8 (7.77)
North Africa	5 (4.85)
Asia	2 (1.94)
East Europe	1 (0.97)
West Europe	2 (1.94)
Americas	2 (1.94)
Other Africa	1 (0.97)
Drug Allergy	
None	82 (79.61)
Penicillin	11 (10.68)
Other Antibiotics	2 (1.94)
Non-Antibiotics	8 (7.77)
Smoking	
Past	13 (12.62)
Never	63 (61.17)
Current	27 (26.21)
PPI treatment, Omeprazole equivalent, mg, mean (SD)	12.5 ± 9.14
Indication	
Dyspepsia	63 (61.17)
GERD	19 (18.45)
Other	21 (20.39)
*H. pylori* treatment history	
Treatment-naïve	64 (62.10)
Treatment-experienced	39 (37.90)
Number of prior attempts, median (range)	1 (1–4)
Time since treatment, years, mean (SD)	4.47 (4.80)

**Table 2:** Endoscopic findings.

Macroscopic Finding	N (%)
Normal	49 (47.50)
Pangastritis	17 (16.50)
Isolated corporal gastritis	6 (5.80)
Isolated antral gastritis	18 (17.50)
Esophagitis	18 (17.40)
Duodenitis	6 (5.80)
Gastric Ulcer	2 (1.90)
Duodenal ulcer	1 (0.97)
Updated Sydney Classification	
Acute Inflammation score, mean (SD)	1.47 (0.81)
Chronic Inflammation score, mean (SD)	1.66 (0.79)
Atrophy score (SD)	1.09 (0.35)
Intestinal Metaplasia score, mean (SD)	1.07 (0.31)
*Helicobacter pylori* score, mean (SD)	0.62 (0.92)

**Table 3:** *H. pylori* test performance *.

	Positive n/N (%)	Sensitivity	Specificity	Accuracy	PPV	NPV	Positive Likelihood Ratio	Negative Likelihood Ratio
Histology	33/103 (32.03)	1.00	0.98	0.99	0.97	1.00	62	0
Rapid urease test	35/98 (35.71)	1.00	0.97	0.98	0.94	1.00	31.5	0
13C-urea breath test	30/100 (30.0)	0.97	1.00	0.99	1.00	0.98	na	0.03
Stool antigen test	22/82 (26.82)	0.70	0.94	0.86	0.86	0.86	18.65	0.31

* Gold standard was defined as concordant RUT and 13C-UBT for histology, concordant histology and 13C-UBT for RUT, and concordant histology and RUT for 13C-UBT. For the stool antigen test, gold standard was defined as the result of at least two of histology, 13C-UBT and RUT.

## Data Availability

Data are available from the corresponding author upon request.
